# Photocatalytic overall water splitting by conjugated semiconductors with crystalline poly(triazine imide) frameworks†
†Electronic supplementary information (ESI) available: Characterization and experimental details. See DOI: 10.1039/c7sc00900c



**DOI:** 10.1039/c7sc00900c

**Published:** 2017-05-30

**Authors:** Lihua Lin, Chong Wang, Wei Ren, Honghui Ou, Yongfan Zhang, Xinchen Wang

**Affiliations:** a State Key Laboratory of Photocatalysis on Energy and Environment , College of Chemistry , Fuzhou University , Fuzhou , Fujian 350002 , P. R. China . Email: xcwang@fzu.edu.cn ; http://wanglab.fzu.edu.cn

## Abstract

Photocatalytic water splitting is an ideal pathway to produce hydrogen for the future energy supply due to the sustainability of solar energy and the mild reaction conditions. In the past four decades, many inorganic semiconductor photocatalysts have been studied for this purpose. In recent years, conjugated polymers, in particular covalent carbon nitride frameworks, have rapidly emerged as a new family of photocatalysts. However, the use of conjugated photocatalysts in overall water splitting in the absence of sacrificial agents has been much less reported. Herein, we used surface kinetic control to photocatalyze overall water splitting by a covalent carbon nitride semiconductor with a crystalline poly(triazine imide) (PTI) frameworks. Our study demonstrates that the loading of a Pt co-catalyst on the PTI surface plays the key role in inducing overall water splitting. The co-deposition of a cobalt species can effectively increase the photocatalytic activity and adjust the ratio of H_2_ and O_2_ produced, as well as enhancing the stability of the photocatalyst. The optimal sample with the dual co-catalysts shows an apparent quantum yield of 2.1% for the overall water splitting reaction.

## Abstract 2

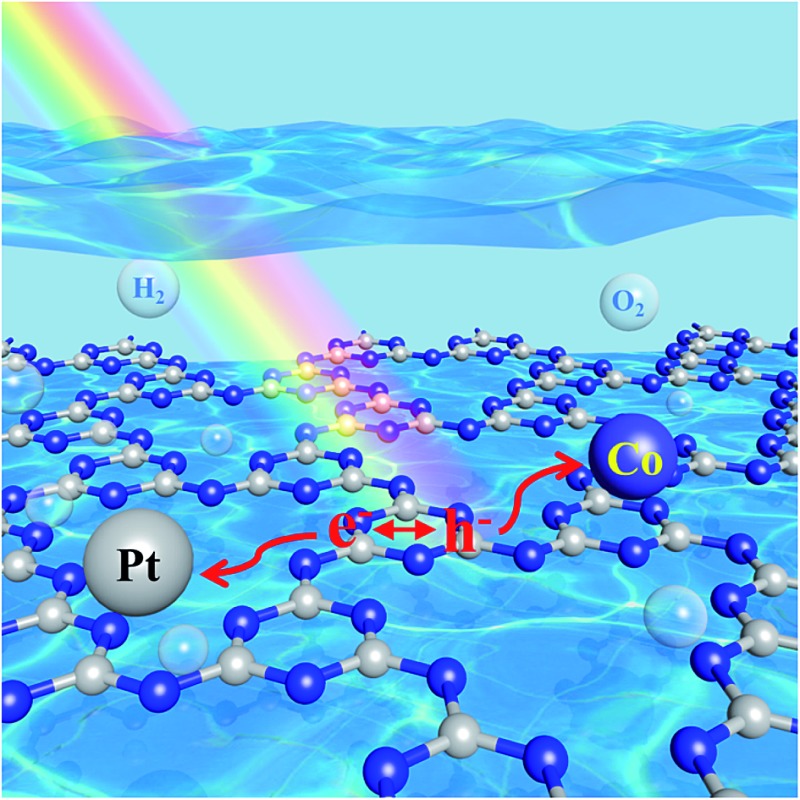
In this work, we apply a carbon nitride semiconductor with a crystalline poly(triazine imide) (PTI) frameworks to photocatalytic overall water splitting.

## Introduction

Photocatalytic water splitting is a fascinating process which can directly cleave water into hydrogen and oxygen gases using abundant solar energy.^1–3^ Regarding the global challenge of energy and the environmental crisis, hydrogen is considered to be a promising candidate for the future energy supply because of its high energy density and it is pollution-free after combustion. Typical overall water photolysis is described as follows: (1) the photocatalyst absorbs a photon with energy equal to or greater than the band gap of the photocatalyst and excites an electron to the conduction band (CB), leaving a hole in the valence band (VB); (2) the electron–hole pair separates and migrates to the catalyst surface; (3) if the photocatalyst possesses suitable CB and VB positions, the electron and the hole can induce the redox splitting of water to produce H_2_ and O_2_.^4–7^

Since 1972, many inorganic semiconductors have been explored for water photolysis, following the pioneering work of Fujishima and Honda.^8–12^ In recent years, conjugated polymers, in particular covalent carbon nitride (CN) semiconductors, have been rapidly developed as a new type of photocatalyst for solar energy utilization.^13–27^ However, the application of polymers in the overall water splitting process is rarely reported, because metal-free polymer photocatalysts typically lack surface active sites with reduced overpotentials for H_2_ or O_2_ evolution. Previous studies have shown that surface kinetic control is an efficient way to create active sites and lower the overpotential of the water cleavage reaction. For example, the loading of a metal or a metal oxide as a co-catalyst can greatly enhance the performance of photocatalysts in overall water splitting.^28–33^ Herein, we used the surface kinetic control method to achieve overall water splitting using a crystalline CN polymer, *i.e.* PTI·HCl, as the heterogeneous photocatalyst.

There are two types of subunit in the CN polymer, *i.e.*, triazine and tri-*s*-triazine (heptazine) motifs.^34,35^ The triazine-based CN possesses a smaller π-conjugated system, indicating a larger band gap than that of the heptazine-based CN. Nevertheless, the triazine-based CN shows higher crystallinity using the current synthesis methods compared to the heptazine-based CN. The representative compound of the triazine-based CN is PTI-based CN. PTI·HCl was the first reported PTI-based CN synthesized using a high-pressure method.^36^ After that, it was found that using the ionothermal method can form the PTI frameworks but it is intercalated with Li and Cl ions, which is typically referred to as PTI/Li^+^Cl^–^.^37,38^ In this study, we adopted the ionothermal method to synthesise a PTI-based CN with high crystallinity. As discussed below, no Li^+^ but Cl^–^ can be found in the final product, which is probably due to the ion-exchange of Li^+^ with H^+^ in the PTI/Li^+^Cl^–^ frameworks. Therefore, we named the sample PTI·HCl. In principle, increasing the crystallinity can facilitate the migration of the photogenerated carriers and reduce the recombination centers.^4,39^ Indeed, the resistivity of PTI·HCl was determined to be ∼300 Ω cm^–1^ using four point probe systems, while no measurable resistivity was determined with a melon-based CN, confirming the enhancement of the carrier conductivity by increasing the crystallinity. Our group has recently achieved overall water splitting with a CN polymer. The as-prepared melon-based CN can activate photocatalytic H_2_ and O_2_ evolution with a stoichiometric ratio of 2 : 1, albeit with a moderate AQY of *ca.* 0.3%, due to poor crystalline frameworks that limit charge separation and migration.^40,41^ Semi-crystalline frameworks typically feature lots of surface defective sites that act as recombination centers for light-triggered electrons and holes. We are for that reason encouraged to apply the highly-crystalline PTI·HCl photocatalyst toward the overall water splitting reaction. Although crystalline PTI-based frameworks have been used for H_2_ production from water splitting, the systems require organic feedstocks (*i.e.* triethanolamine, methanol and ethanol) as sacrificial agents to complete the water redox splitting cycle. To the best of our knowledge, overall water splitting by crystalline PTI-based photocatalysts without using any sacrificial agent has not yet been reported, thus far.

## Results and discussion

**Fig. 1:**
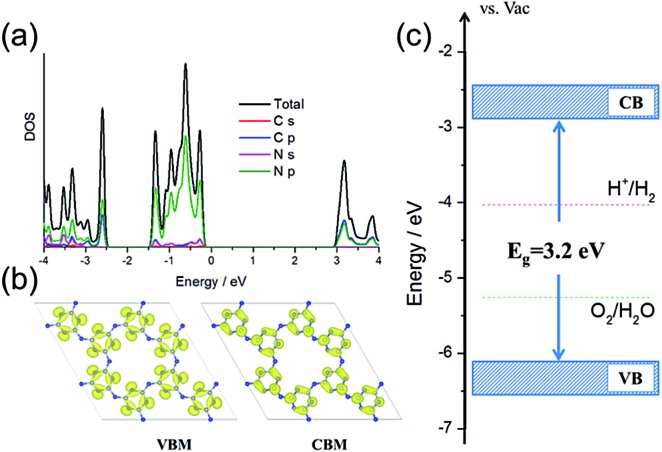
(a) The total and projected DOS of the PTI. (b) The band decomposed charge density of the VBM and CBM of the PTI. (c) The band alignment of the PTI *vs.* the redox levels of water.

First, density functional theory (DFT) calculations were carried out to get insight into the electronic properties and band structure of PTI·HCl. As shown in Fig. 1a and b, the density of states (DOS) and band decomposed charge density confirmed that the VB of the PTI was dominated by N 2p states and the CB was hybridized by C 2p and N 2p states, which were similar to those of the melon-based CN.^42–44^ Fig. 1c shows that the CB and VB edges of the PTI are straddling the water redox levels, which thermodynamically ascertains that the overall water splitting is energetically favorable. Furthermore, the calculated VB edge is lower than that of the melon-based CN with respect to the vacuum level (Fig. S1†), indicating the higher oxidation ability of PTI·HCl.^13^ This is critical for the overall water splitting reaction, which is limited by water oxidation *via* a four-electron process. Overall, the calculated results provide evidence that PTI·HCl is a potential photocatalyst to achieve overall water splitting to produce H_2_ and O_2_.

**Fig. 2:**
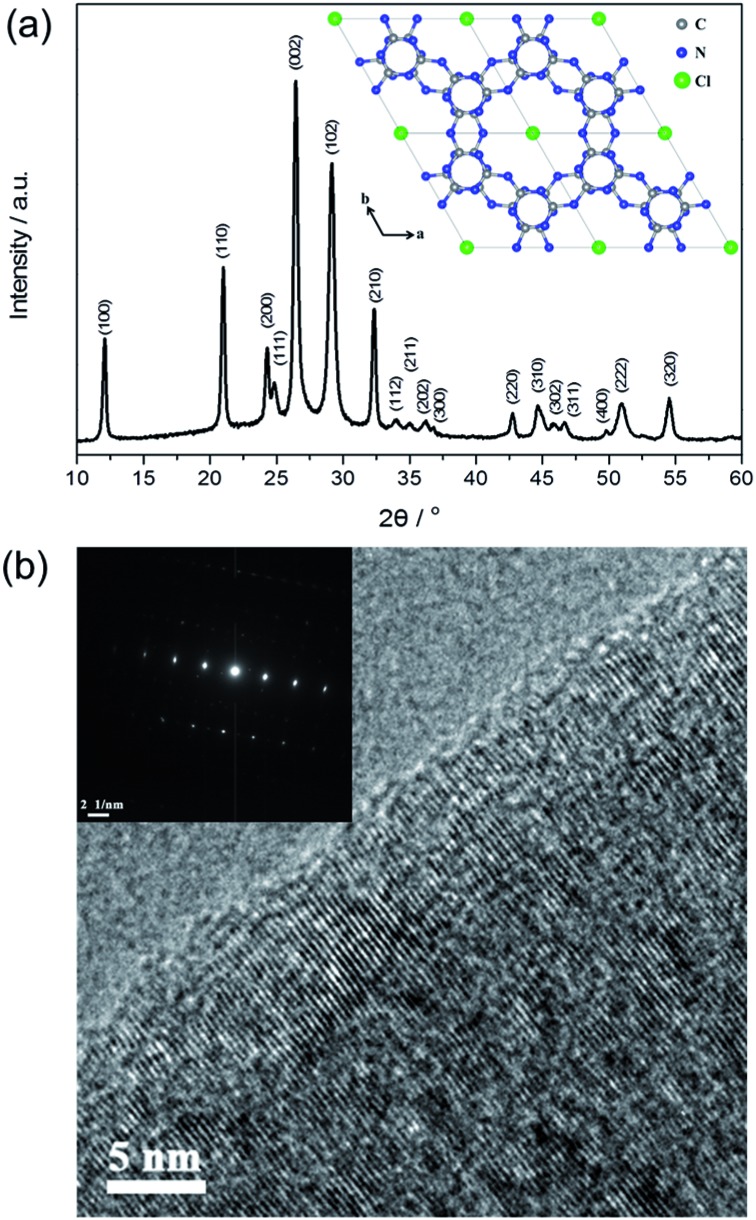
(a) The XRD pattern of PTI·HCl. Inset: the structure model of PTI·HCl (H atoms are removed for clarity). (b) The high resolution TEM image of PTI·HCl. Inset: the SAED pattern of the (002) plane.

Fig. 2a shows the X-ray diffraction (XRD) pattern of PTI·HCl. Several intense peaks are observed in the spectrum, indicating good crystallization of the sample. The structure of PTI·HCl is determined as a hexagonal unit cell with the space group *P*6_3_ *cm* (no. 185). The lattice parameters are *a* = *b* = 8.45218 Å and *c* = 6.73827 Å, which are consistent with those in the previous study.^37,38^ The strongest peak is located at 26.4°, corresponding to an interlayer distance of 3.37 Å, which can be indexed as (002) facets. The PTI·HCl structure has been widely investigated in previous studies, which have demonstrated that PTI·HCl is constructed from the infinite linkage of triazine tectons *via* NH bridges to form 2D planes with Cl ions intercalated between the layers (Fig. 2a inset).^45–47^ Therefore, the well-resolved structure of PTI·HCl offers a good model for the investigation of the photocatalytic properties of CN polymers.

The morphology of PTI·HCl synthesized by the ionothermal method was investigated by scanning electron microscopy (SEM) and transmission electron microscopy (TEM). Regular cube and hexagonal prisms are generally observed in a large scale (Fig. S2†). The morphology of PTI·HCl is significantly different from that of the melon-based CN prepared by the thermal-induced polymerization method, which typically shows stacking of the bulk particles.^48–50^ Hence, the ionothermal method is an efficient pathway to mediate the morphology-evolution of the CN polymers. The cube and hexagonal prisms are observed in the TEM images as well (Fig. S3a and b†), similarly to the SEM results. The high resolution TEM images show clear lattice fringes in the sample (Fig. 2b and S3c and d†). The selected area electron diffraction (SAED) pattern of the (002) facets exhibits well-defined stacking of the graphite-like layers perpendicular to the c-axis with an interplanar spacing of 3.37 Å (Fig. 2b inset). The TEM analyses together with the XRD results indicate good crystallization of PTI·HCl.

The chemical composition of the as-prepared PTI·HCl was investigated by X-ray photoelectron spectroscopy (XPS). Four sharp peaks are observed in the survey spectrum, relating to C, N, Cl and O elements (Fig. S4a†). The high resolution C 1s spectrum can be decomposed into two peaks at 287.7 eV and 284.8 eV (Fig. S4b†). The former intense peak is attributed to the sp^2^-hybridized graphitic carbon atoms in the N-contained triazine rings (N–C=N), while the latter one probably originates from adventitious carbon (such as grease). The N 1s spectrum is deconvoluted into two peaks (Fig. S4c†). The peak at 398.3 eV is due to pyridine-like N involved in the triazine rings (C–N=C). The other peak at 399.8 eV is typically attributed to the bridge N.^51–53^ The signal of the chlorine atoms shows a relatively high intensity (Fig. S4d†). On the contrary, no obvious peaks for Li and K can be detected in the high resolution spectra (Fig. S4e and f†), which is probably due to the substitution of Li^+^ with H^+^ *via* ion-exchange during the massive washing with water.^36^ Therefore, we refer to the sample as PTI·HCl for chemical balance.

Optical absorption is an important parameter for photocatalysts. In principle, strong and long-wavelength optical absorption would be favorable for the photocatalytic reaction due to the high utilization ratio of the incident light. The UV-vis diffuse reflectance spectra (DRS) pattern of PTI·HCl shows semiconductor-like absorption with an absorption edge at around 400 nm (Fig. S5†). The band gap of the as-prepared PTI·HCl is estimated to be ∼3.1 eV. Long wavelength absorption is observed with a relatively low absorption intensity for PTI·HCl, which is distinct from the sample synthesized with a relatively short calcination time. This is probably due to the formation of a small amount of triazine-based graphitic carbon nitride, which can absorb >600 nm light.^54^ The FTIR spectrum shows the vibrational fingerprint of the triazine units at 810 cm^–1^ (Fig. S6†). Broad peaks around 1200–1600 cm^–1^ belong to the stretching and bending vibrations of the triazine heterocycles. The peaks around 3310 and 3200 cm^–1^ originate from the amino groups.^37,55^ The ^13^C direct excitation nuclear magnetic resonance (NMR) spectrum of PTI·HCl shows three peaks that are attributed to the triazine units (Fig. S7†), which is consistent with the previous literature.^38^ The N_2_ adsorption isotherms of PTI·HCl and the melon-based CN show an increased surface area of PTI·HCl (36 m^2^ g^–1^) compared to the melon-based CN (12 m^2^ g^–1^) by thermal-induced polycondensation (Fig. S8†).

**Fig. 3:**
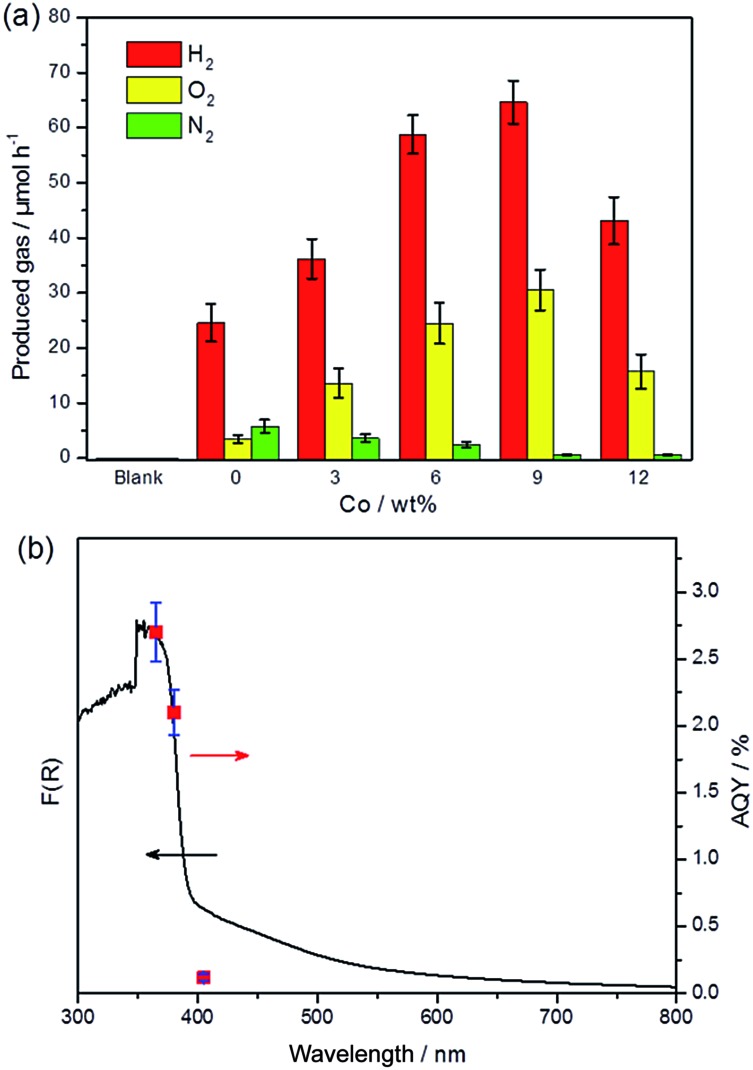
(a) Photocatalytic overall water splitting of PTI·HCl using the full arc irradiation of a 300 W Xe lamp. All of the samples were co-loaded with 1 wt% Pt as a co-catalyst except for the blank one. (b) Wavelength-dependent AQY of PTI·HCl loaded with 1 wt% Pt and 9 wt% Co.

Photocatalytic overall water splitting was performed in a Pyrex top-irradiation reaction vessel using pure water. The photocatalytic activities at the initial stage are shown in Fig. 3a. The pure PTI·HCl (blank) sample exhibited no detectable activity toward overall water splitting. However, hydrogen and oxygen gases were detected when *in situ* photodeposition of 1 wt% Pt occurred on the PTI·HCl surface. This result demonstrates that the loading of Pt is a critical step to induce the water splitting reaction, although the stoichiometric ratio of hydrogen and oxygen was not equal to 2 : 1 and nitrogen gas was also detected. Note that the oxygen evolution rate is slower than the theoretical value if the hydrogen evolution rate is used as the reference. Therefore, the photocatalyst plays the role of the sacrificial agent to consume the photogenerated holes and lead to the partial decomposition of PTI·HCl.^56^

To address this issue, the promotion of the oxidation side of the water redox splitting reaction is desired, and therefore cobalt was loaded as the co-catalyst to accelerate the oxygen evolution reaction. The typical procedure for Co loading is the impregnation method, by which Co(NO_3_)_2_ is mixed with a photocatalyst and then calcinated at a certain temperature in air to form CoO_*x*_.^57^ However, applying this method to load Co onto PTI·HCl leads to a decrease in the activity, which is probably due to the partial decomposition of PTI·HCl in the presence of metal ions during calcination (Fig. S9†). To solve this problem, we applied an *in situ* photochemical method to deposit the Co species onto the surface of PTI·HCl as the oxygen evolution co-catalyst. Remarkably, the *in situ* photodeposition of the Pt/Co co-catalysts onto PTI·HCl leads to a dramatically enhanced performance and it inhibits the release of N_2_. The evolution rates of H_2_ and O_2_ increase with the amount of Co to a maximum at 9 wt%, beyond which the activity of the samples begins to decrease. Furthermore, the loading of 1 wt% Pt and 9 wt% Co on the PTI·HCl surface also shows a stoichiometric ratio of H_2_ and O_2_ approximately equal to 2 : 1. Only a trace amount of N_2_ was detected. Therefore, proper deposition of the Co species can not only increase the photocatalytic activity but also stabilize the photocatalyst against photocorrosion. Furthermore, the melon-based CN only shows relatively lower hydrogen and oxygen production with a non-stoichiometric ratio under the same conditions (Fig. S10†), which highlights the important role of crystallinity in overall water splitting.

To further confirm that the photocatalytic water splitting reaction was driven by the absorption of an incident photon, the wavelength-dependent photocatalytic water splitting experiment was also performed using different monochromatic light. As shown in Fig. 3b, the apparent quantum yield (AQY) values matched well with the DRS pattern before ∼400 nm. However, the AQY value shows clear deviation from the DRS pattern at 405 nm. As mentioned previously, the extended absorption of the sample is mostly likely to originate from the formation of a small amount of the triazine-based graphitic carbon nitride, which may show inactivity toward overall water splitting. Additionally, the amount of H_2_ and O_2_ significantly decreased when the light was turned off (Fig. S11†). In contrast, the amount of H_2_ only slightly decreases after turning the light off in photocatalytic hydrogen production (Fig. S12†). These results indicate the obvious backward reaction in overall water splitting. Therefore, further investigation is required in order to suppress the backward reaction. The XRD patterns of PTI·HCl before and after the photocatalytic reaction confirm the stability of the photocatalyst during the prolonged operation (Fig. S13†).

**Fig. 4:**
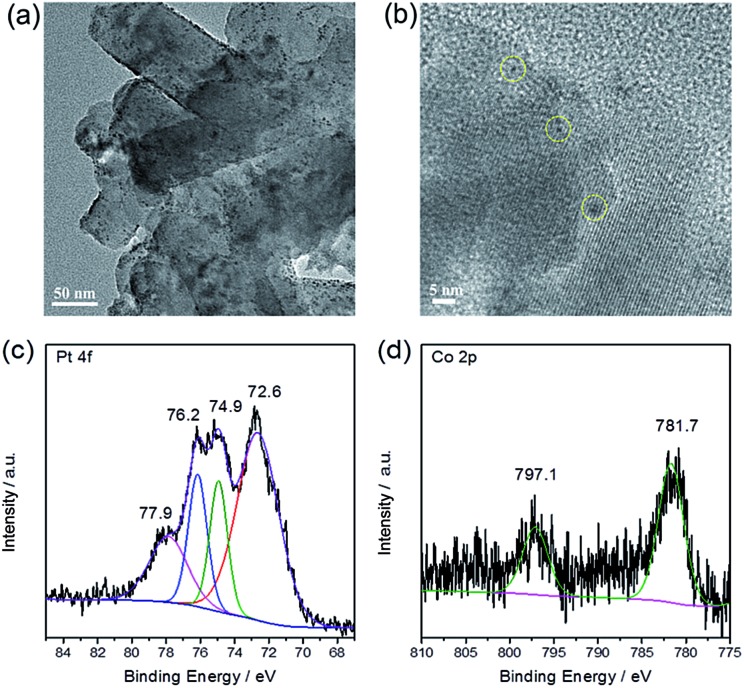
(a and b) The TEM images of Pt/Co loaded PTI·HCl. The yellow dotted circles indicate the Pt or CoO_*x*_ nanoparticles. High-resolution XPS spectra of (c) Pt 4f and (d) Co 2p using the *in situ* photodeposition method.

Finally, the morphology and chemical state of the loaded Pt/Co were investigated by TEM, EDX and XPS. Fig. 4a and b show the TEM images of Pt/Co loaded PTI·HCl, indicating the successful deposition of Pt/Co nanoparticles on the photocatalyst surface. The lattice fringe of Pt was determined as 0.23 nm (Fig. S14†). In contrast, for the Co species, the lattice fringe was not observed, probably due to the amorphous structure or low crystallinity of the Co species. Therefore, EDX analysis was carried out. The Pt/Co together with C, N and Cl elements were observed in the EDX spectrum, further confirming the loading of the Pt and Co species (Fig. S15†). Fig. 4c shows the high resolution XPS spectra of Pt, which can be deconvoluted into two pairs of peaks, assigned to Pt^0^ and Pt^2+^. Previous work has demonstrated that Pt^0^ is the effective hydrogen evolution co-catalyst and that PtO_*x*_ can promote the evolution of oxygen.^40^ The high resolution XPS spectra of Co, shown in Fig. 4d, confirmed the formation of the CoO_*x*_ species, which has been widely investigated as a co-catalyst for oxygen evolution.^58–60^ To further study the roles of the co-catalysts, we have carried out additional tests. As shown in Fig. S16,† both the loading of Co or Pt on PTI·HCl can prompt the hydrogen evolution reaction. Interestingly, the co-loading of Pt/Co shows a synergistic effect in the enhancement of the activity toward hydrogen production. As for the oxygen evolution half reaction, the loading of Co or PtO_*x*_ on PTI·HCl shows obvious enhanced performance (Fig. S17†). Therefore, the co-loading of Pt/Co co-catalysts can effectively create catalytically active centres to accelerate H_2_ and O_2_ production. In addition, it is of note that the oxygen evolution rate in the water oxidation half reaction was lower than that in overall water splitting, which was due to the loading of Ag onto the photocatalyst surface, leading to the shielding of active sites and incident light.

## Conclusions

In summary, we have demonstrated an efficient pathway to achieve photocatalytic overall water splitting using a single PTI·HCl photocatalyst with a crystalline conjugated frameworks to act as a highway to shuttle charge migration. The loading of Pt plays a key role in inducing the water photolysis, although the produced H_2_ and O_2_ are non-stoichiometric and N_2_ was detected due to the photocorrosion of the photocatalyst. The *in situ* photodeposition of Pt/Co on the photocatalyst gives an enhanced photocatalytic activity and one can adjust the ratio of the produced H_2_ and O_2_ to 2 : 1, as well as the stabilization of the photocatalyst. Our results highlight the important role of surface kinetic control in the overall water splitting process. This system offers a good platform to investigate the overall water splitting reaction in a single metal-free crystalline CN polymer photocatalyst. Further modifications on both the bulk and the surface may improve the absorption ability in the visible light region and prevent the backward reaction, which is promising for the utilization of solar energy using sustainable and abundant carbon nitride materials in relevant reactions including water splitting, CO_2_ fixation and organic synthesis.
