# Supplementary material for: Photocatalytic overall water splitting by conjugated semiconductors with crystalline poly(triazine imide) frameworks
Source: Chem Sci. 2017 May 30;8(8):5506–11. doi: 10.1039/c7sc00900c (PMC5613792; doi:10.1039/c7sc00900c)
Supplement: Supplementary file 1 [file SC-008-C7SC00900C-s001.pdf]

Electronic Supplementary Material (ESI) for Chemical Science.
This journal is © The Royal Society of Chemistry 2017

Supporting Information

Experimental Section

Materials. Dicyandiamide(DCDA, $C_2H_4N_4$, 99%) was purchased from Acros. KCl (99%) and LiCl (99%) were purchased from Alfa Aesar Chemicals Co. Ltd. (China). All the materials were used without further purification.

Synthesis of Samples. 1g DCDA was ground with KCl (6.6g) and LiCl (5.4g) in glove box. Then, the mixture was transferred to an open glass tube and heated to 400°C. After cooling to room temperature, the glass tube was sealed and heated to 600°C for 24 hours at a rate of $1^{o}C \cdot min^{-1}$ in a tube furnace. After cooling to room temperature, the sample was washed with boiling deionized water several times and dried at 60°C under vacuum. This sample was referred to as PTI·HCl. Melon-based CN was synthesized by direct heating 1g DCDA to 550 °C without using KCl/LiCl.

Characterization. X-ray diffraction (XRD) measurements were performed on a Bruker D8 Advance diffractometer with Cu-Kα_1 radiation (λ=1.5406 Å). The solid-state ^{13}C MAS nuclear magnetic resonance (NMR) spectra were recorded on a Bruker Advance III 500 spectrometer. The Fourier transform infrared (FTIR) spectra were recorded on a BioRad FTS 6000 spectrometer. The UV-Vis diffuse reflectance spectra (DRS) were performed on a Varian Cary 500 Scan UV-visible system. The X-ray photoelectron spectroscopy (XPS) data were obtained using a Thermo ESCALAB250 instrument with a monochromatized Al Kα line source (200 W). The morphologies of the samples were investigated using HITACHI SU8010 field emission scanning electron microscopy (SEM). Transmission electron microscopy (TEM) and energy dispersive X-ray spectroscopy (EDX) was performed on a FEI Tencai 20 microscope. The nitrogen adsorption-desorption isotherms were collected at 77 K using a Micromeritics ASAP 2020 surface area and porosity analyzer. Resistivity was carried out by using RTS-4 four point probe systems. Electrochemical characterizations were performed using a BioLogic VSP-300 electrochemical system, using saturated calomel electrode (SCE) as the reference electrode and Pt plate as the counter electrode. The working electrode was prepared by electrophoretic deposition on FTO substrates. The 20 mg sample was suspended in 30 mL acetone containing 10 mg I_2 via ultrasonication. The electrophoretic deposition of PTI·HCl was carried out using a Pt plate (10×10× 0.2 mm) as an anode. Deposition was performed for 1 min at 40 V. After air drying, the working electrode was annealed at 250 °C for 30 min to improve adhesion. The measurement was performed in a 0.2 M Na_2SO_4 aqueous solution (pH=6.8).[1]

Cocatalysts deposition. The Co or Pt cocatalyst was loaded on the photocatalyst surface by *in situ* photodeposition method. Briefly, 100 mg photocatalyst powder was dispersed in deionized water (100 mL) contained 10% vol (10 mL) methanol (MeOH) as the sacrificial agent. A certain amounts of $CoCl_2$ (3 wt%, 6 wt%, 9 wt%, 12 wt%, based on Co atoms) or H_2PtCl_6 (1 wt%, based on Pt atoms) were added into the solution. After 1 hour photodeposition using full arc irradiation of 300W lamp, the photocatalyst was filtration and washed with deionized water several times. Then the photocatalyst was dried at 60 °C for 4 hours.

The Co and Pt cocatalysts were loaded on the photocatalyst surface by in situ photodeposition method. Briefly, 100 mg photocatalyst powder was dispersed in deionized water (100 mL) contained 10% vol (10 mL) MeOH as the sacrificial agent. A certain amounts of $CoCl_2$ (3 wt%, 6

wt%, 9 wt%, 12 wt%, based on Co atoms) were added into the solution. After 1 hour photodeposition using full arc irradiation of 300W lamp, the photocatalyst was filtration and washed with deionized water several times. Then, the photocatalyst powder was dispersed in deionized water (100 mL) contained 10% vol (10 mL) methanol as the sacrificial agent. A certain amounts of H_2PtCl_6 (1 wt%, based on Pt atoms) were added into the solution. After 1 hour photodeposition using full arc irradiation of 300W lamp, the photocatalyst was filtration and washed with deionized water several times. Then the photocatalyst was dried at 60 °C for 4 hours.

The PtO_x/PTI·HCl was synthesized by a typical immersion strategy followed by thermal treatment in the air. Typically, 200 mg of PTI·HCl powders was immersed in 5 mL of deionized water water followed ultrasonication for 5 minutes. Then, a certain amounts of H_2PtCl_6 (1 wt%, based on Pt atoms) were added into the solution. The final resultant sample was obtained after evaporation and thermal treated in muffle furnace at 300 °C for 1 h.

Photocatalytic activity test. The reactions were carried out in a Pyrex top-irradiation reaction vessel connected to a glass closed gas system. The hydrogen production was performed by dispersing 100 mg of photocatalyst powder in pure water (100 mL) contained 10% vol (10 mL) MeOH as the sacrificial agent. The oxygen production was performed by dispersing 100 mg of photocatalyst powder in pure water (100 mL) contained 0.01 M $AgNO_3$ as the sacrificial agent and 0.2g La_2O_3 as a pH buffer agent. The overall water splitting was carried out by dispersing 100 mg of photocatalyst powder in pure water (100 mL). The reaction solution was evacuated several times to completely remove the air prior to full spectrum irradiation of 300 W Xe lamp. The temperature of the reaction solution was maintained at room temperature using a flow of cooling water during the reaction. The evolved gases were analyzed by a gas chromatography equipped with a thermal conductive detector (TCD) and a 5A molecular sieve column, using argon as the carrier gas.

The apparent quantum yield (AQY) for the overall water splitting was determined by replacing the Xe lamp with LEDs equipped with different band-pass filters. The irradiation area was 9 cm^2. The total intensity irradiation was measured by averaging 10 points in the irradiation area. For example, the average intensity was 6.8 mW cm^{-2} for the 380nm monochromatic light (ILT 950 spectroradiometer). The AQY was calculated as follow:

$$AQY=N_e/N_p\times100\%=2M/N_p\times100\%$$

where N_e is the amount of reaction electrons, N_p is the incident photons, M is the amount of H_2 molecules.

Theoretical calculations. The periodic DFT calculations were performed using pure PBE[2] XC functional and the plane wave basis sets[3] as implemented in the Vienna *ab initio* simulation package (VASP).[4-6] The C s^2p^2, N s^2p^3 were treated as valence electrons. The cutoff energy for the plane-wave basis set was 550 eV. The geometry optimizations were performed using the conjugate gradient technique, until the total energies converged to 10^{-4} eV and the Hellmann–Feynman forces on the atoms were less than 0.01 eV $Å^{-1}$. For band calculations, the high symmetry k-paths were obtained from the literature.[7] The single layer model of melon-based CN was used in present calculation. Although some programs have been developed, the theoretical calculation of random structures remains challenging. Therefore, we only constructed the backbone of PTI·HCl with single layer as the preliminary model. In this regard, further investigation is required to reveal the influence of those ions on the electronic properties and

bandstructure of PTI·HCl.

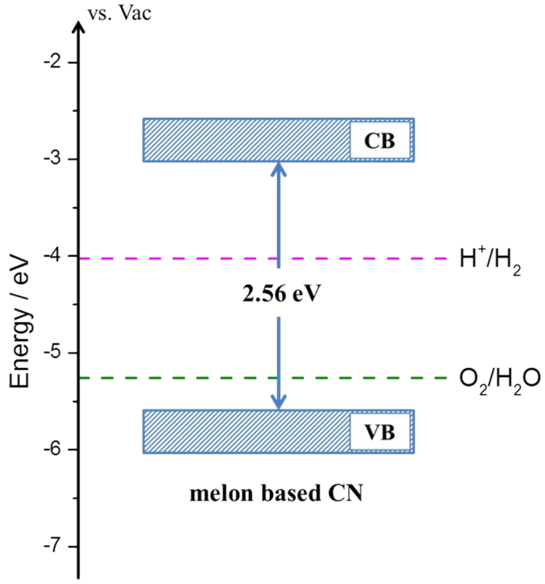

Figure S1. The band alignment melon-based CN vs. redox levels of water.

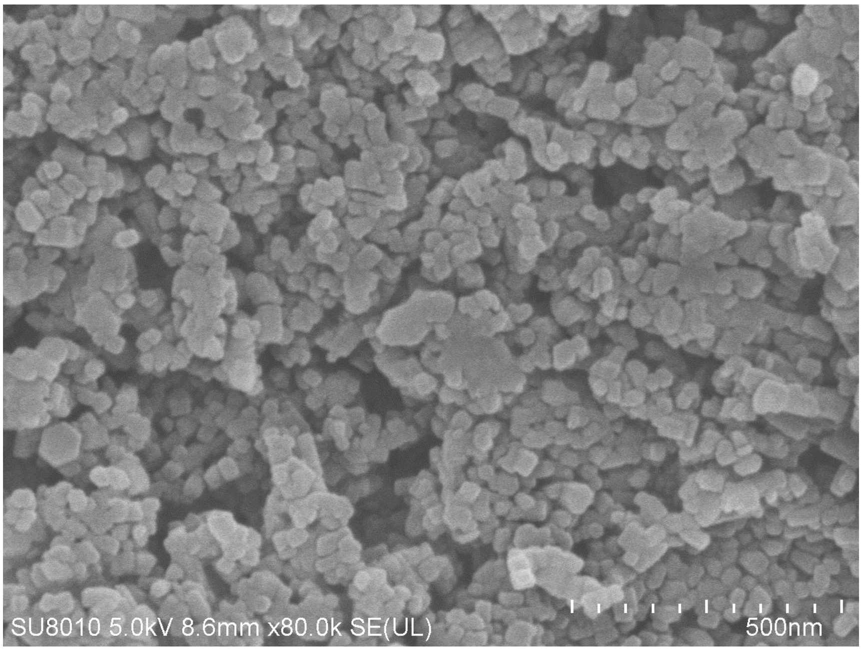

Figure S2. The SEM image of PTI·HCl.

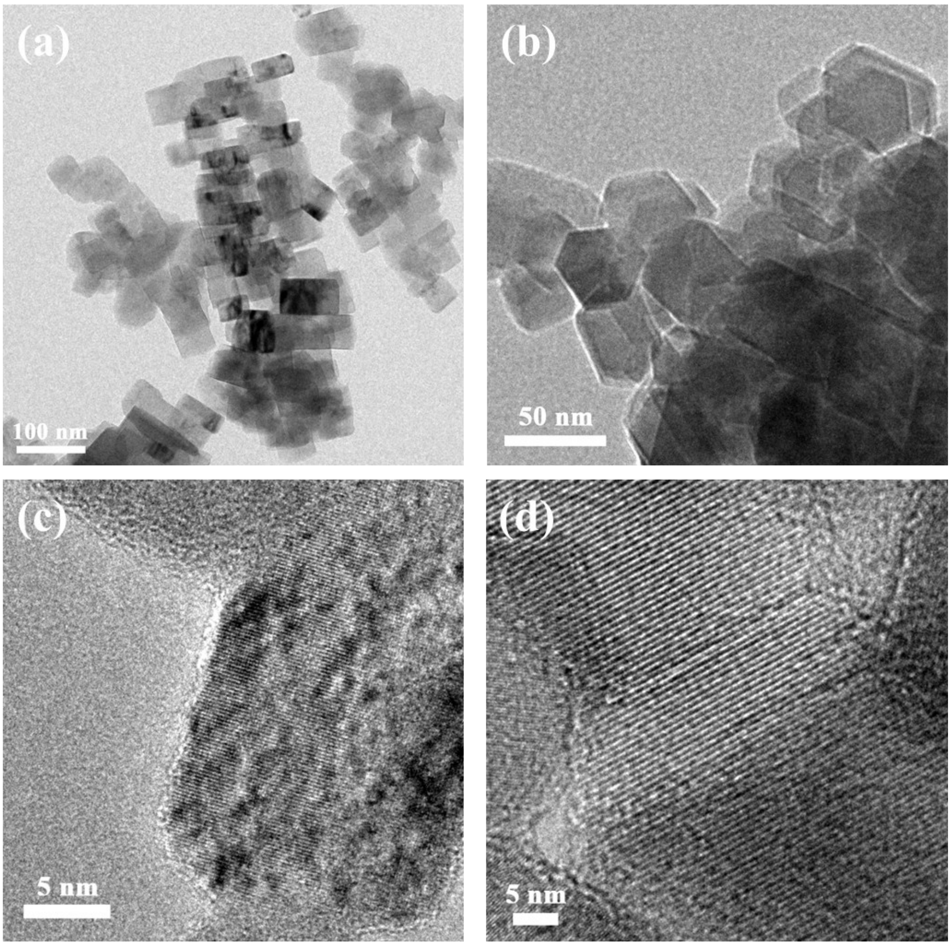

Figure S3. (a, b) The TEM image of PTI·HCl. (c, d) The HR-TEM images of (110) and (100) plane.

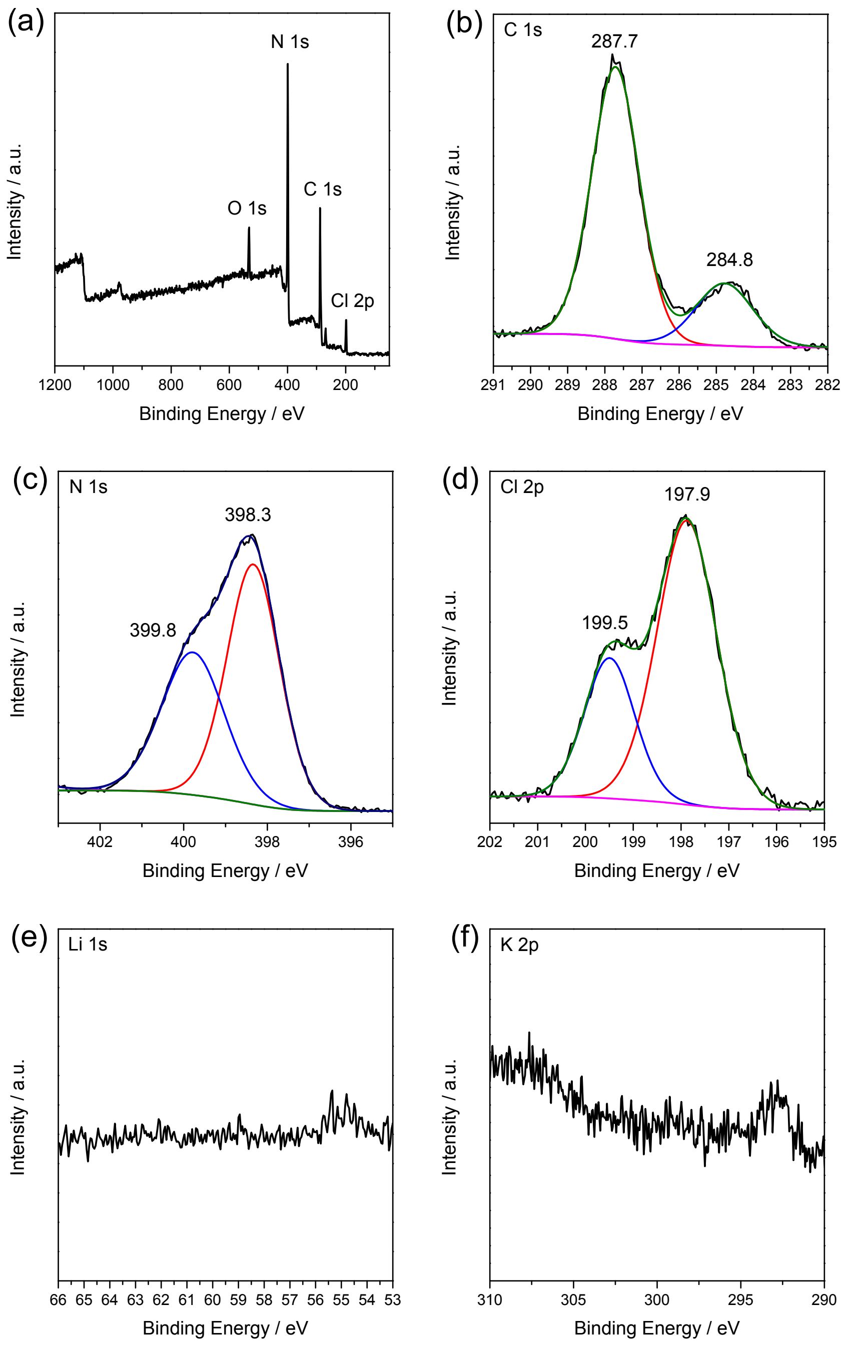

Figure S4. XPS results of (a) the survey spectrum and high-resolution spectra of (b) C 1s, (c) N 1s, (d) Cl 2p, (e) Li 1s and (f) K 2p for PTI·HCl.

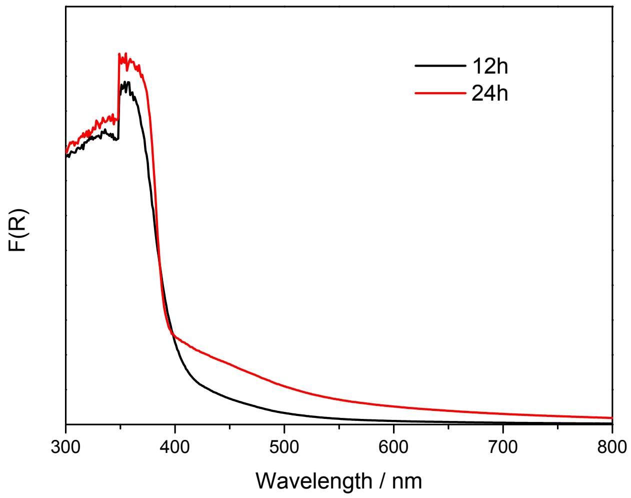

Figure S5. DRS of PTI·HCl.

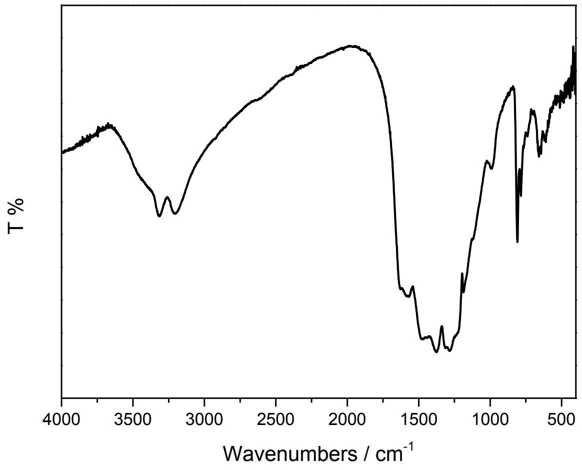

Figure S6. FTIR spectrum of PTI·HCl.

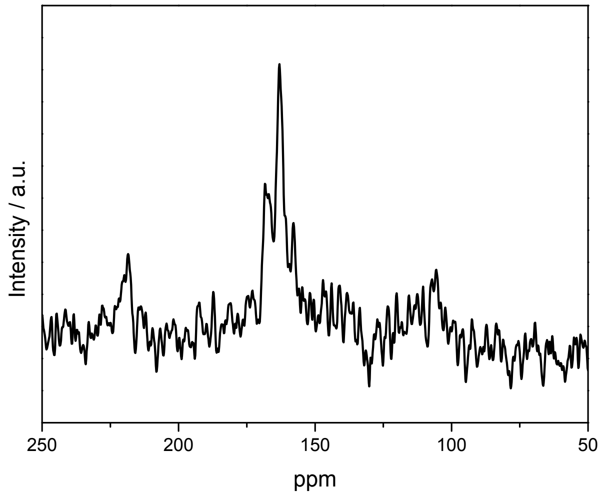

Figure S7. ^{13}C direct excitation NMR spectrum of PTI·HCl.

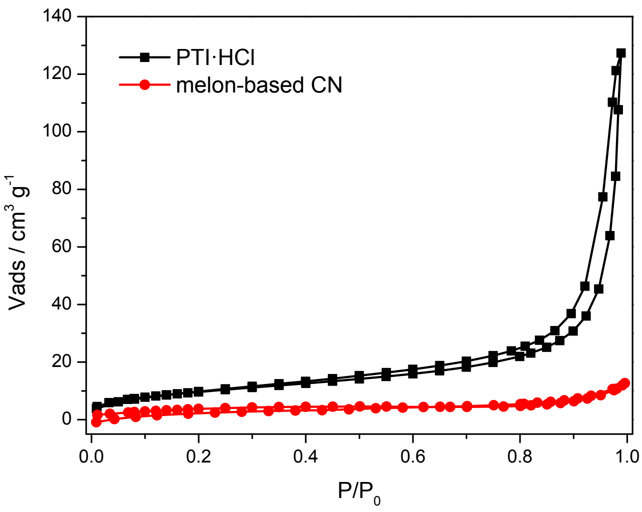

Figure S8. N_2 adsorption and desorption isotherms collected at 77 K of PTI·HCl and melon-based CN.

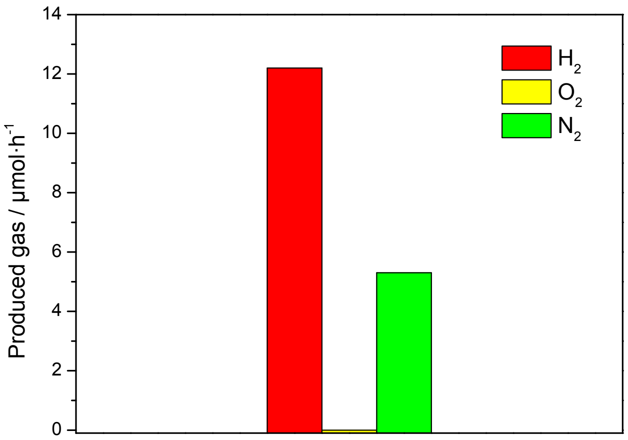

Figure S9. The photocatalytic activity of Pt/Co loaded PTI·HCl, which Co species was loaded using impregnation method.

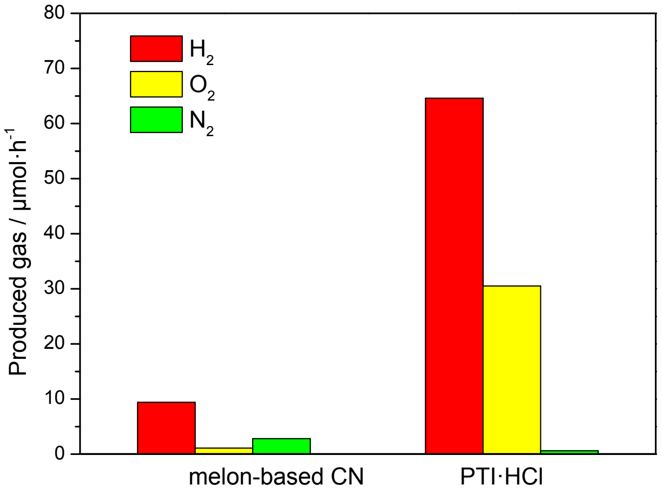

Figure S10. The overall water splitting of melon-based CN and PTI·HCl.

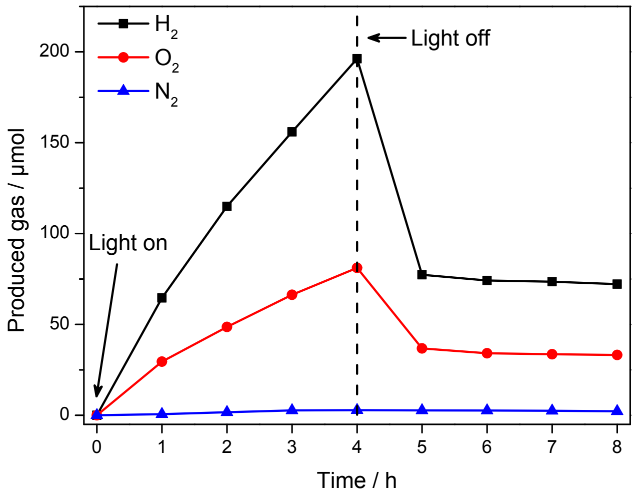

Figure S11. Overall water splitting of 1 wt. % Pt and 9 wt% Co modified PTI·HCl under full arc irradiation of 300W Xe lamp and in dark.

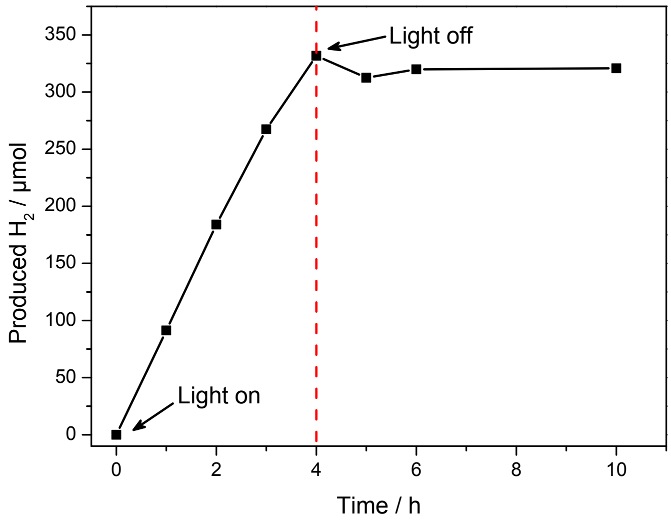

Figure S12 The hydrogen production of PTI·HCl under full arc irradiation of 300W Xe lamp and in dark using MeOH as the sacrificial agent.

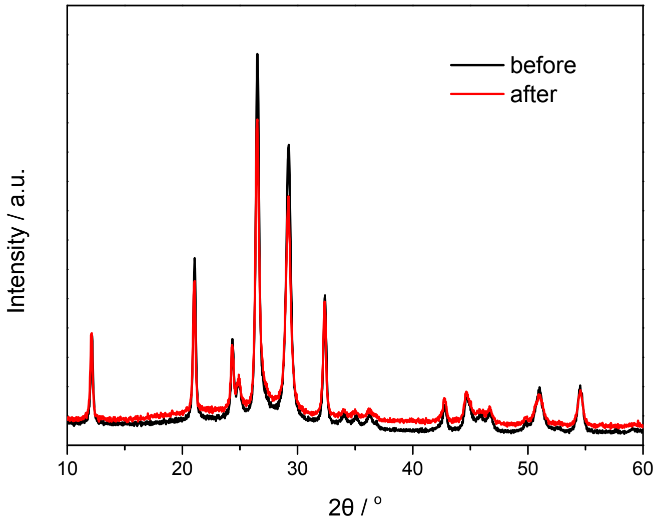

Figure S13. The XRD pattern of PTI·HCl before and after photocatalytic reaction.

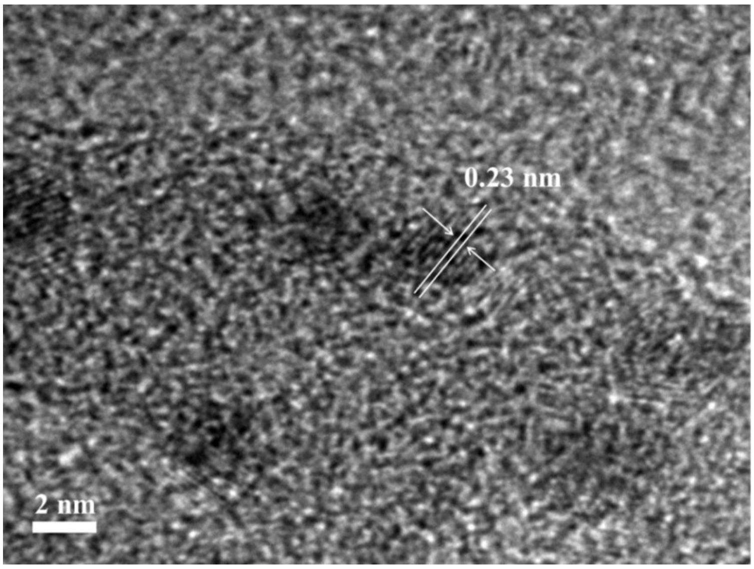

Figure S14. The TEM image of Pt/Co loaded PTI·HCl.

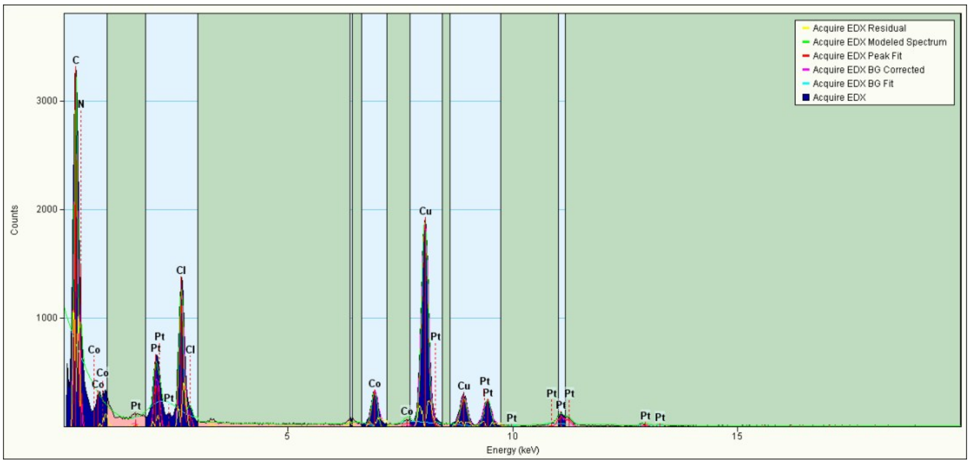

Figure S15. The EDX analysis of Pt/Co loaded PTI·HCl.

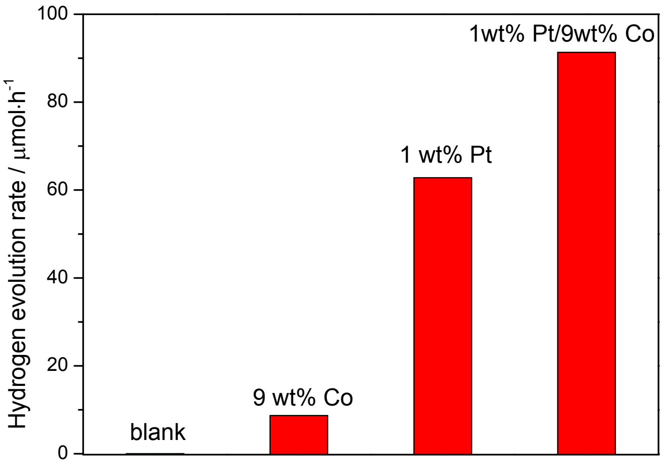

Figure S16. The hydrogen evolution rate of PTI·HCl and PTI·HCl loading with different cocatalyst using MeOH as the sacrificial agent.

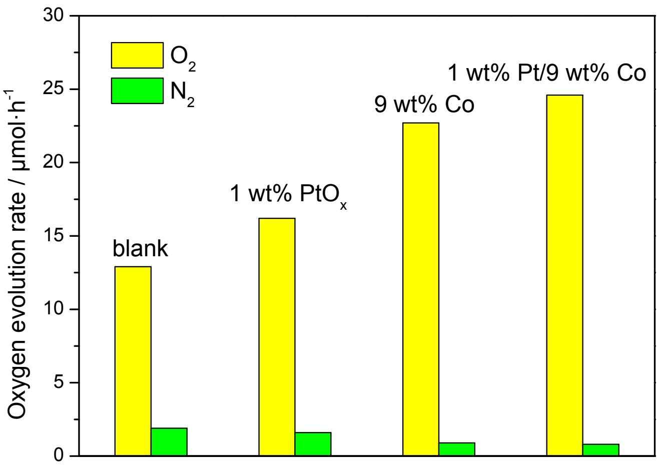

Figure S17. The oxygen evolution rate of PTI·HCl and PTI·HCl loading with different cocatalyst using $AgNO_3$ as the sacrificial agent.

Reference

1 W.-J. Chun, A. Ishikawa, H. Fujisawa, T. Takata, J. N. Kondo, M. Hara, M. Kawai, Y. Matsumoto and K. Domen, *J. Phys. Chem. B*, 2003, **107**, 1798-1803.

2 J. P. Perdew, K. Burke and M. Ernzerhof, *Phys. Rev. Lett.*, 1996, **77**, 3865-3868.

3 P. E. Blöchl, *Phys. Rev. B*, 1994, **50**, 17953-17979.

4 G. Kresse and J. Furthmüller, *Phys. Rev. B*, 1996, **54**, 11169-11186.

5 G. Kresse and J. Furthmüller, *Comput. Mater. Sci.*, 1996, **6**, 15-50.

6 G. Kresse and D. Joubert, *Phys. Rev. B*, 1999, **59**, 1758-1775.

7 W. Setyawan and S. Curtarolo, *Comput. Mater. Sci.*, 2010, **49**, 299-312.
